# Unraveling the paternal genetic structure and forensic traits of the Hui population in Liaoning Province, China using Y-chromosome analysis

**DOI:** 10.1186/s12864-023-09774-8

**Published:** 2023-11-17

**Authors:** Dazhi Fu, Atif Adnan, Jun Yao, Noura H. Aldayan, Chuan-Chao Wang, Cao Hongyi

**Affiliations:** 1 First Affiliated Hospital of China Medical University, 155 Heping District, Shenyang, 110001 China; 2 Department of Forensic Sciences, Collage of Criminal Justice, Naif Arab University for Security Sciences, Riyadh, Saudi Arabia; 3 Department of Forensic Biology and Genetics, School of Forensic Medicine, China Medical University, Shenyang, 110001 China; 4 Department of Medical Laboratory Sciences, College of Applied Medical Sciences, Prince Sattam Ibn Abdulaziz University, Al-Kharj, 16273 Saudi Arabia; 5 Department of Anthropology and Ethnology, Institute of Anthropology, School of Sociology and Anthropology, Xiamen University, Xiamen, Fujian People’s Republic of China; 6 Department of Pathology, School of Basic Medical Sciences, China Medical University, Shenyang, 110001 China

**Keywords:** Hui people, Y-SNPs and STRs, Liaoning province, Paternal genetic makeup, Forensic traits

## Abstract

The Hui people are the second-largest ethnic minority in China, and they are distributed throughout the country. A previous study explored the paternal genetic structure of the Hui population in nine different regions of China, but it overlooked the Liaoning province. In this study, we examined the paternal genetic makeup and forensic traits of the Hui population in Liaoning province by analyzing 157 Y-chromosome single nucleotide polymorphisms (Y-SNPs) and 26 short tandem repeats (Y-STRs). We successfully genotyped 282 unrelated male individuals from the Hui population of Liaoning province using the SNaPshot® single base extension assay and Goldeneye™ Y26 system kit (PEOPLESPOT R&D, Beijing, China). The results revealed high haplotypic diversity (0.9998) and identified 46 terminal haplogroups for the Hui population. Additional analyses, such as heat maps, principal component analysis (PCA), genetic distance (FST), Multidimensional scaling (MDS) analysis, and median-joining network (MJ) analysis, showed that the Hui population could be classified into three groups: Northwest Hui populations (NWH), including Liaoning, Xinjiang, Qinghai, Gansu, Ningxia, Shaanxi, and Henan; Hui populations from Sichuan and Shandong (SSH); and Yunnan Hui populations (YNH). Pairwise genetic distance (Rst) comparisons with other Chinese populations revealed that the Hui population displayed genetic affinity with the Han population. The comprehensive understanding of the Hui population in Liaoning province, explored by Y-SNPs and Y-STRs, can be utilized to interpret their genetic structure and enhance the accuracy of forensic databases.

**Supplementary Information:**

The online version contains supplementary material available at 10.1186/s12864-023-09774-8.

## Introduction

Short tandem repeats (Y-STRs) and single nucleotide polymorphisms (Y-SNPs) on the male-specific Y chromosome are valuable genetic markers used in population genetics and forensic investigations. These markers have distinctive inheritance patterns that enable the identification of paternal lineages and the reconstruction of male ancestry [1, 2]. Y-STRs and Y-SNPs are applied in population genetics to explore the demographic history of human populations, including migration trends, mixing, and divergence [3–5]. They are also crucial in forensic investigations for identifying male suspects and victims when DNA evidence is limited or degraded, particularly in sexual assault cases where male DNA profiles can be obtained even in mixtures with female DNA [6, 7]. Moreover, Y-STRs and Y-SNPs are essential for understanding the evolutionary and biological processes that have affected the Y chromosome throughout time, in addition to their practical uses [4, 8]. These markers have been utilized to investigate the rates and patterns of mutation on the Y chromosome, as well as the genetic basis of male fertility [9–11]. In a previous study [12], researchers explored the paternal genetic structure of the Hui population in nine different regions of China. The Hui population is the third largest group in China after Han and Zhuang, with a population size of over 10.5 million. They settled across China but mainly in Ningxia (34.77%), Qinghai (14.83%), Gansu (11.89%), Yunnan (6.60%), Shandong (5.06%), and Liaoning (0.632%) (http://www.stats.gov.cn/). The ancestors of the Hui came to China from Central Asia and Islamic Persia as handicraftsmen, merchants, scholars, and soldiers. This human movement started in the seventh century and continued until the thirteenth century. After settling in China, they intermingled with Han Chinese, Mongols, and Uyghurs, ultimately assimilating their phenotype, cultural characteristics, and language into Chinese culture. In Liaoning province, the Hui population represents only 0.632% of the population and is the fourth largest ethnic group after Han (83.94%), Manchu (12.88%), and Mongol (1.60%) [13]. Previous studies have indicated that the Hui population from Ningxia has a closer relationship with other East Asian populations [14]. Another study explored the Y-STRs of the Xinjiang Hui population, revealing genetic affinity with Han and Xibe ethnic groups [15]. Our previous study [16] showed a close association between the Hui population from Xinjiang and the Han population. Other studies have focused on autosomal insertion deletion markers in the Xinjiang Hui population and X-STRs in the Ningxia Hui population, yielding results in accordance with Y-STR studies [17, 18]. However, few studies related to the Hui population residing in Liaoning province of China are available in terms of Y-STRs in forensic casework analysis and population genetics studies, and those that do exist have been conducted using a limited number of Y-STRs [19, 20]. In this study, we used the SNaPshot® single base extension assay and the Goldeneye Y26 System (Peoplespot, Beijing China) kit to provide a more precise reference database of the Hui population from Liaoning province for forensic investigation and population studies.

## Materials and methods

### Sample collection and ethical approval

This study was approved by the Ethical Review Board of China Medical University in Shenyang, Liaoning Province, People's Republic of China, in accordance with the standards of the Declaration of Helsinki. Blood samples on FTA cards were randomly collected from 282 unrelated healthy male individuals of the Hui population living in Liaoning province for at least three generations. The aims and procedures of the study were explained to all volunteers, and they signed informed consent forms before participating.

### DNA extraction and quantification

DNA was extracted from bloodstains collected on FTA cards (Changchun Bokun Biotech CO., Ltd, China) using the Chelex-100 method [21]. DNA quantification was performed using the QuantifilerTM Human DNA Quantification Kit (Applied Biosystems, Foster City, CA, USA) according to the manufacturer's instructions.

### PCR amplification and genotyping

A total of 157 Y-SNPs were selected from elsewhere [22], representing major haplogroups CT, C, DE, D, F, G, H, I, J, K, LT, L, N, O, P, Q, R, and their corresponding sub-haplogroups. These inhouse developed assays of 157 YSNP markers were then categorized into one basic and five high-resolution SNaPshot assays, following a hierarchical genotyping approach (Supplementary Table 1). The genotyping process began with a single multiplex PCR, performed in a 15 μl volume. The PCR mixture contained 1–2 ng of template DNA, 7.5 μl of Multiplex PCR Mix (Qiagen, Hilden, Germany), and 0.1 μM of primers. The maximum amplicon size allowed was 230 base pairs. The PCR was carried out using a GeneAmp PCR System 9700 (Applied Biosystems, Foster City, CA) under the following conditions: pre-incubation at 95°C for 10 min, followed by 30 cycles of denaturation at 94°C for 30 s, annealing at 60°C for 90 s, extension at 72°C for 60 s, and a final extension step at 72°C for 30 min.

Next, the PCR products were purified by adding 1 μl of exonuclease I and 2 μl of shrimp alkaline phosphatase (SAP) (TaKaRa Biotechnology (Dalian) Co., Ltd) to a 9 μl multiplex PCR product. The mixture was then incubated at 37°C for 60 min, followed by enzyme denaturation at 80°C for 10 min [23]. This reaction was carried out in a 10 μl volume, containing 2.5 μl of SNaPshot ready reaction mix, 0.5 μl PCR Gold buffer (Applied Biosystems, Foster City, CA), 1 μl of purified PCR product, and 0.014–0.213 μM of primers. The thermal cycling conditions for the SNaPshot reaction involved 25 cycles of denaturation at 96°C for 10 s, annealing at 50°C for 5 s, and extension at 60°C for 30 s.

Following the SNaPshot reaction, the reaction products were purified by adding 1.25 μl of SAP and incubating at 37°C for 60 min, followed by enzyme denaturation at 80°C for 10 min. Further analysis was performed by mixing 1 μl of the purified SNaPshot reaction product with 9.5 μl of Hi-Di formamide and 0.50 μl of LIZ-120 internal sizing standard (Applied Biosystems, Foster City, CA). The mixture was then subjected to denaturation at 95°C for 3 min, followed by cooling to 4°C. The samples were separated on an ABI Prism 3100 Genetic Analyzer (Applied Biosystems, Foster City, CA) using a 36 cm capillary array and the default instrument settings recommended by the manufacturer. The Goldeneye™ 26Y system kit (PEOPLESPOT R&D, Beijing, China), which contains PowerPlex Y23 loci and three additional Y-STRs (DYS388, DYS449, and DYS460), were co-amplified in a GeneAmp® PCR 9700 (Life Technologies, CA, USA) thermal cycler according to the manufacturer's recommendations [24]. The 2800M (Promega Corporation, USA) was used as a positive control in all batches, and a PCR negative control was included in each batch. Amplified PCR products were detected and separated with reference to the ORG 500 internal size standard (Goldeneye™) and Goldeneye™ 26Y Allelic Ladder using an ABI 3100 genetic analyzer (Applied Biosystems, Foster City, CA) in accordance with the Goldeneye™ 26Y amplification system kit (PEOPLESPOT R&D, Beijing China.) recommendations.

### Statistical analysis

#### Y-SNP data analysis

Haplogroup frequencies were calculated by direct counting and presented in pie charts using Microsoft Excel. Haplotype diversity was calculated according to the formula:

$$\frac{n\left(1 - \sum {\mathrm{pi}}^{2}\right)}{\left(\mathrm{n }-1\right)}$$

where pi represents the frequency of the i*th* haplogroup, and n is the sample size. Heat map matrices were performed based on haplogroup frequency in the R program, and the ggplot2 module was used. Arlequin 3.5.2 [25] was used to calculate the analysis of molecular variance (AMOVA) and pairwise genetic distances of FST within the Hui population. Principal component analysis (PCA) was performed based on haplogroup frequencies using SPSS 19.0 software (SPSS Inc., USA).

#### Y-STR data and forensic genetic parameter analysis

Allelic frequencies and haplotypes were calculated by the direct counting method, while haplotype diversity (HD) was calculated using the formula:

$$\mathrm{HD }= \frac{n}{n -1} \left(1 - {\sum }_{i}{p}_{i}^{2}\right)$$

where n is the male population size, and pi is the frequency of the ith haplotype. Haplotype diversity (HD) was estimated at six different levels which includes 9 Y STR loci of minimal haplotype (MH) (which includes DYS19, DYS389I, DYS389II, DYS390, DYS391, DYS392, DYS393 and DYS385a/b) [26], 11 loci of extended haplotype (EH) (MH Y STRs + DYS438 and DYS439) [27], Powerplex Y 12 (EH Y STRs + DYS437) [28], Yfiler™ (PPY 12 STRs + DYS448, DYS456, DYS458, DYS635 and GATA_H4) [29], Powerplex Y 23 (Yfiler STRs + DYS481, DYS533, DYS549, DYS570, DYS576 and DYS643) [30], Goldeneye™ 26Y system kit (PPY 23 STRs + DYS388, DYS460 and DYS449) [24]. The discrimination capacity (DC) was calculated as the number of different haplotypes divided by the total number of samples. Pairwise genetic distance (Rst) and associated probability values (p-values, 10,000 permutations) were calculated using the AMOVA on the YHRD website [31]. Reduced dimensionality spatial representation of the populations based on Rst values was performed using multi-dimensional scaling (MDS) with IBM SPSS Statistics for Windows, Version.

#### Analysis of Y-SNP and Y-STR data

The median-joining networks was constructed using NETWORK 5.0.0.3 [32]`. We set the weighting according to our previous study (Hazara Study). The intermediate alleles were rounded to the nearest number. Duplicate marker (DYS385) was removed from analysis and any null allele was replaced with “99” in input files.

## Results and discussion

### Genetic diversity

The genetic diversity of the study population, consisting of 282 self-identified Hui individuals from Liaoning, China, and their genotypic profiles are summarized in Supplementary Table 2. All individuals had unique profiles at 26 Y STRs, resulting in a random matching probability (RMP) of 0.0035. The number of total haplotypes reduced to 273 when using the commonly used Yfiler 17 Y STRs, with 264 (93.61%) being singletons. The haplotype diversity (HD) was 0.9998 ± 0.0003, the discrimination capacity (DC) was 0.9680, and the RMP was 0.0038. When reducing the number of STRs from 26 to 9, the total number of haplotypes was 260, with 242 (85.81%) being unique. The HD was 0.9993 ± 0.0004, and the DC was 0.9219. A summary of all these results is presented in Table 1. Supplementary Table 3 presents the allele frequencies, allele number per locus, and respective gene diversities. Among the single copy allele STRs, the gene diversity (GD) ranged from 0.3905 (DYS391) to 0.8783 (DYS449), while for multi-copy loci (DYS385), the gene diversity was 0.9659. The allele frequencies ranged from 0.0035 to 0.7021. DYS385 was the most polymorphic allele, with 59 allele combinations, while DYS437 and DYS460 were the least polymorphic, with only 4 alleles.

**Table 1:** Forensic parameters at 6 different levels in Hui population from Liaoning on Y STRs

	**9 Y STRs**	**11 Y STRs**	**12 Y STRs**	**17 Y STRs**	**23 Y STRs**	**26 Y STRs**
Random Matching Probability (RMP)	0.0042	0.0039	0.0039	0.0038	0.0035	0.0035
Haplotype diversity (HD)	0.9993 ± 0.0004	0.9996 ± 0.0003	0.9996 ± 0.0003	0.9998 ± 0.0003	1.0000 ± 0.0003	1.0000 ± 0.0003
Total Haplotypes (TH)	260	267	267	273	282	282
Unique haplotypes (UH)	242	253	253	264	282	282
No of pairs haplotypes (PH)	14	13	13	9	0	0
No of trio haplotypes (tH)	4	1	1	0	0	0
Discrimination capacity (DC)	92.19%	94.68%	94.68%	96.80%	100%	100%
% of Unique (UH%)	85.81%	89.71%	89.71%	93.61%	100%	100%

### Haplogroup distribution

**Fig. 1:**
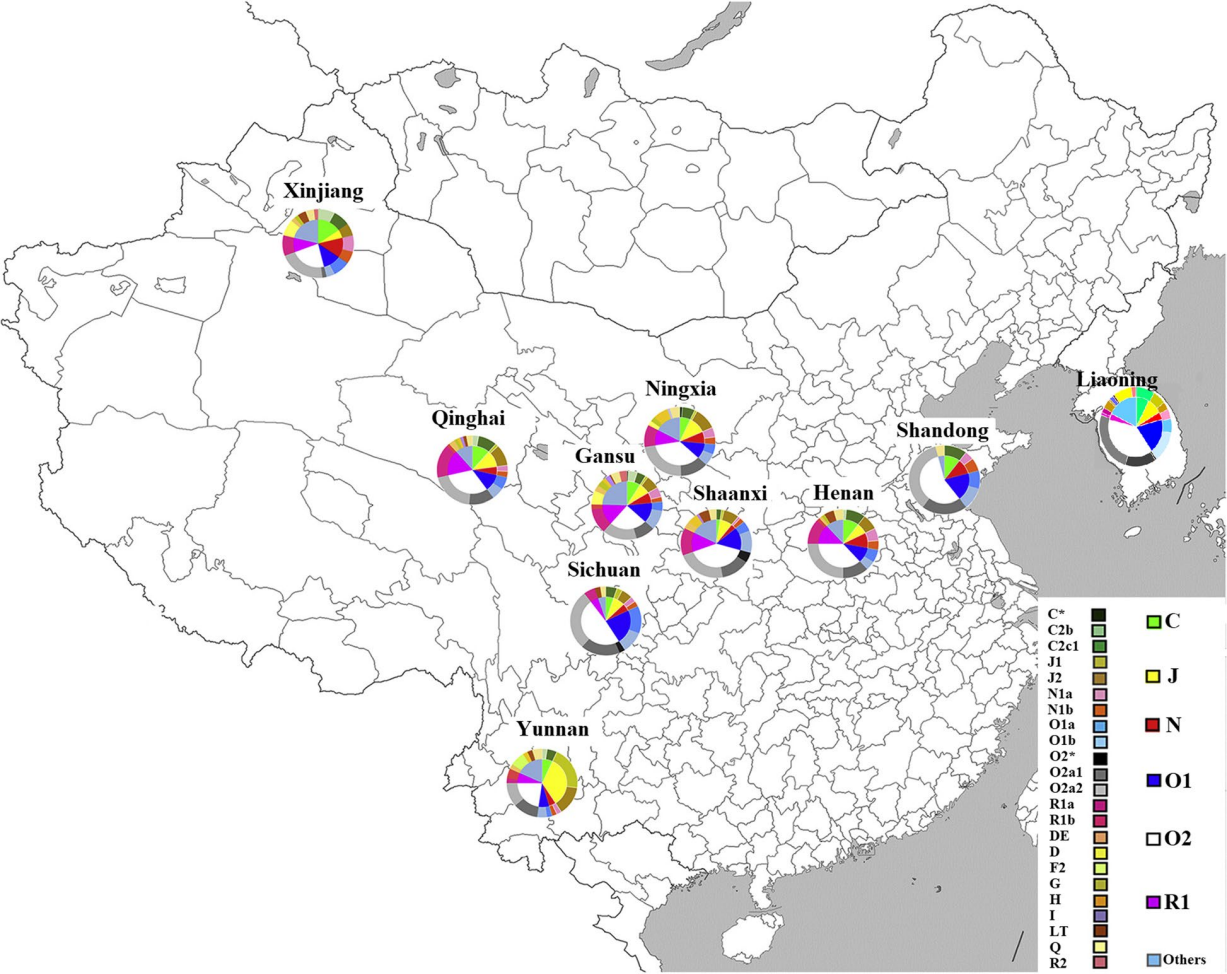
Haplogroup distributions in the ten different geographical regions of China

The Hui population from Liaoning showed high haplogroup diversity with 46 terminal groups. The predominant haplogroups were O (57%), J (8.86%), C (8.51%), Q (8.15%) and D (5.31%) in Hui population from Liaoning. When we have combined our data with previously published data of Hui population [12], 99 distinct terminal haplogroups discovered. (Supplementary Fig. 1). The predominant haplogroups in the Hui people were O (47.38%), R (11.85%), J (9.69%), C (8.31%), and N (7.54%). This indicates that a significant portion of the Hui population shares a common genetic ancestry. Haplogroup C2c1, D1a1a1a, N1 and O2a1c1a1a1a1a1a1a were only found in Liaoning Hui population. Haplogroup D1a was found erratically in Liaoning, Xinjiang, Gansu, and Ningxia which shows the Tibetan influence [33]. This suggests that there may have been genetic differentiation or isolation among Hui communities in different regions, leading to the development of unique haplogroup distributions. Only Hui population from Yunnan showed the presence of F2 haplogroup. These regional variations in haplogroup distribution highlight the diversity within the Hui population and the impact of geographical and historical factors on their genetic makeup. Overall, haplogroup O2 showed more dominance in comparison to haplotype O1 in Hui populations from across China. Heat map based on the presence of the terminal haplogroup showed us that Hui population across China can be divided into three major groups: those from the northwest (NWH), those from Sichuan and Shandong (SSH), and those from Yunnan (YNH). Significant differences in major haplogroup frequencies were observed at the regional and sub-regional levels (Fig. 1). Which implies that there are notable variations in the distribution of haplogroups among Hui populations in different geographic areas and within smaller sub-regions. These differences could be attributed to various factors such as historical migrations, cultural exchanges, and genetic drift within isolated communities. Further research on these variations could provide insights into the genetic history and ancestry of the Hui people.

### Genetic differences along the landscape of China among Hui population

We have calculated the pairwise genetic distances Fst (Supplementary Table 4) and analysis of molecular variance (AMOVA) (Supplementary Table 5) between Liaoning Hui and other Hui populations from Gansu, Xinjiang, Henan, Ningxia, Qinghai, Sichuan, Shandong, Shaanxi and Yunnan to analyze the genetic relationship. According to Fst genetic distance, population from Shandong (0.0025) showed the closest affinity followed by Sichuan (0.0035) population while population from Yunnan (0.0096) showed the largest genetic distances which was followed by Gansu (0.0075).

**Fig. 2:**
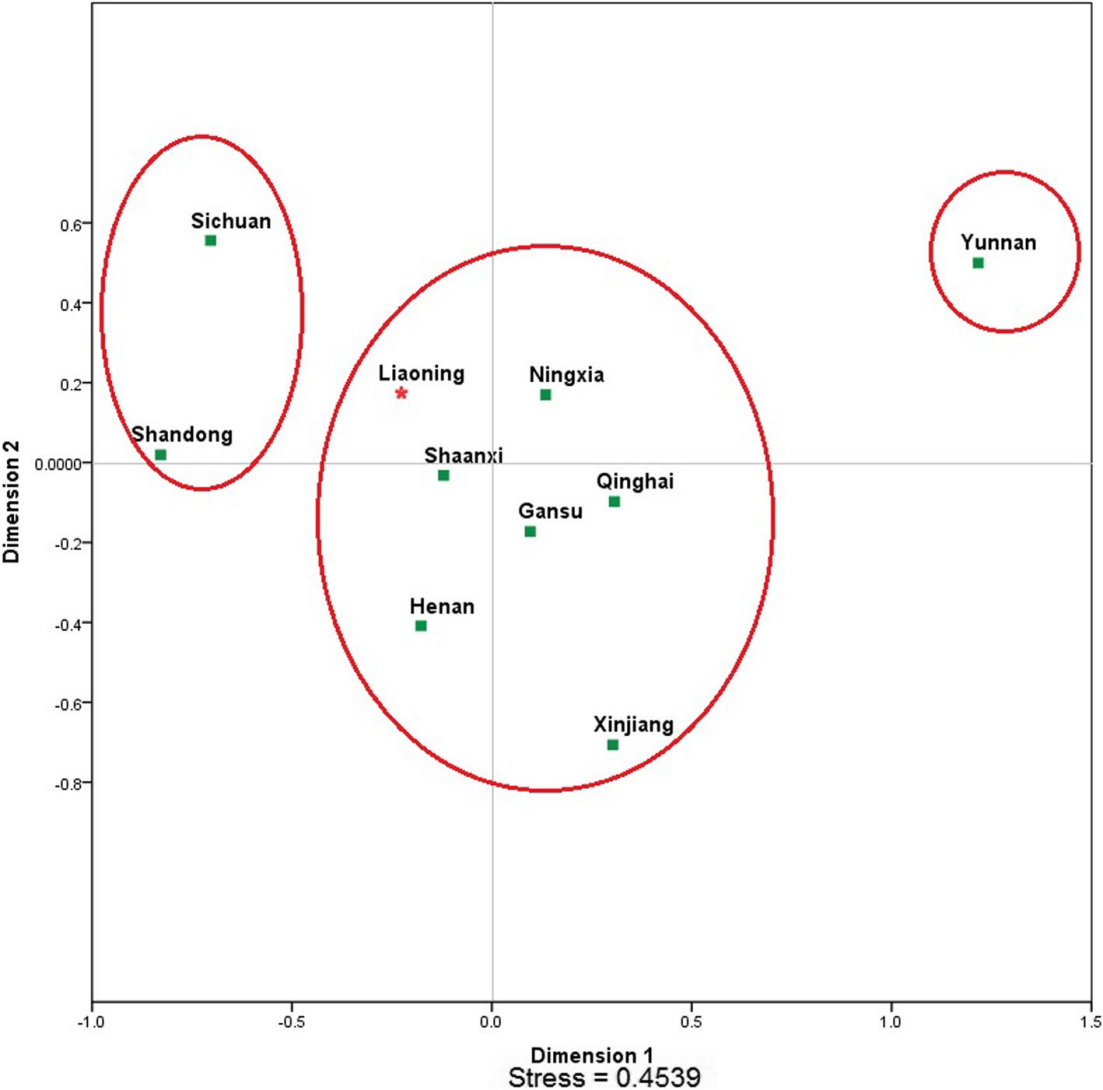
Two-dimensional plot from multi-dimensional scaling analysis of Fst-values based on PPY-23 haplotypes for 10 Hui populations

**Fig. 3:**
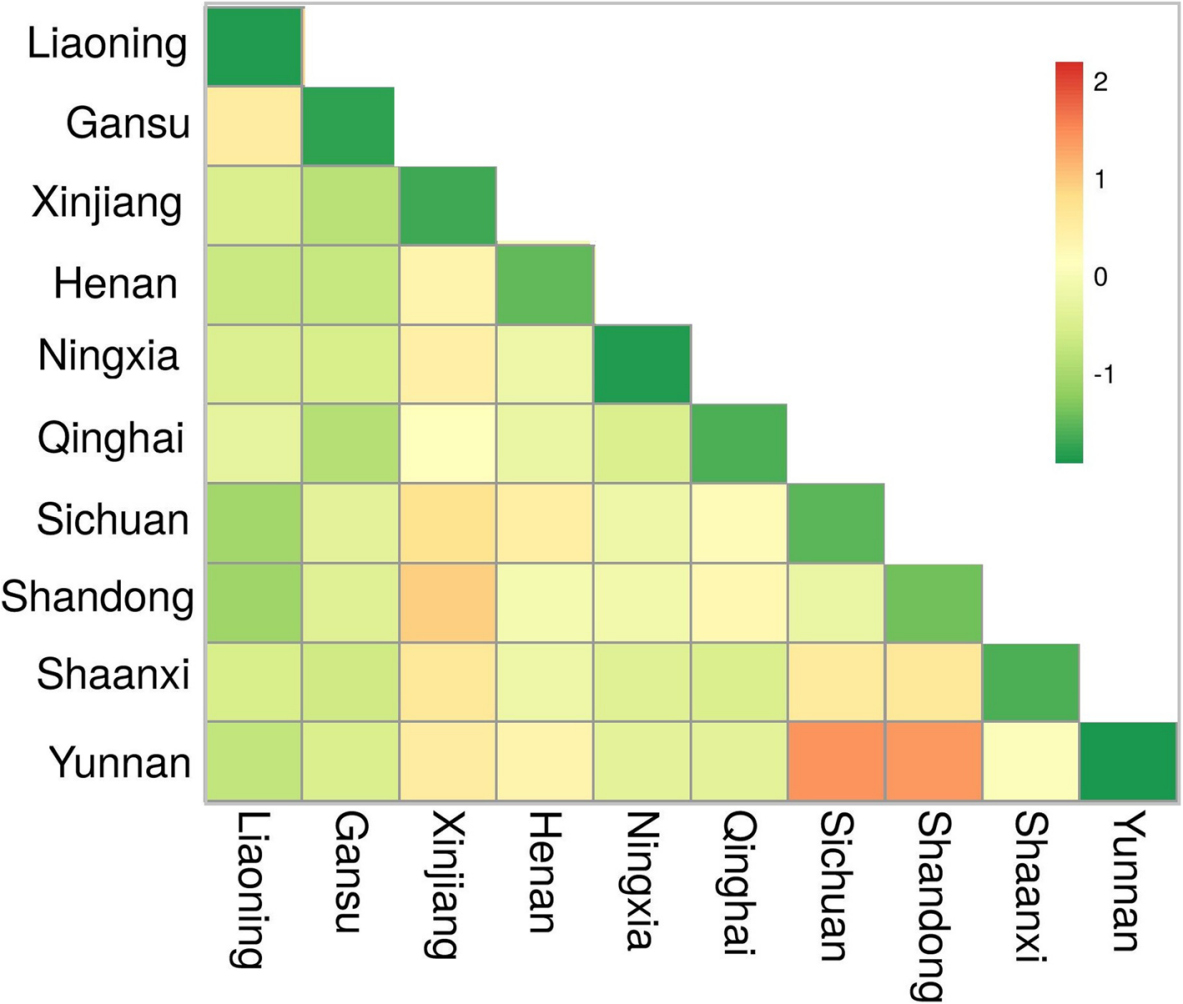
Heat map of pairwise FST between 10 Hui populations based on 157 Y-SNPs

**Fig. 4:**
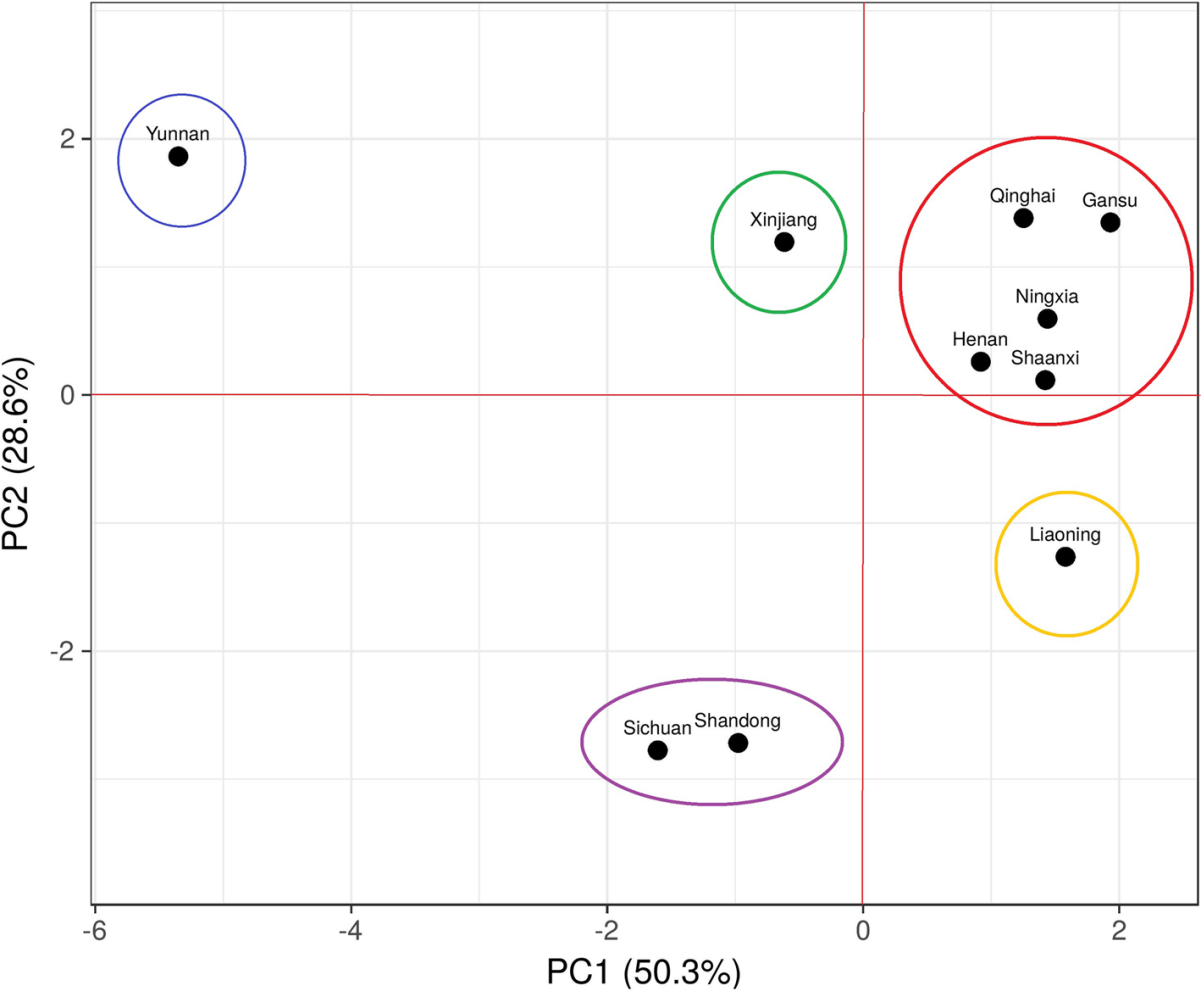
Principal component analysis between 10 Hui populations

In MDS plot among 10 Hui populations (Fig. 2) from different regions of China, we have found 3 different clusters. On left, upper side we found a cluster of Shandong Hui and Sichuan Hui while in the middle of the plot we found 7 hui groups from Liaoning, Shaanxi, Henan, Ningxia, Gansu, Qinghai and Xinjiang. Yunnan Hui population formed a separate cluster on the upper right side of the plot. Results of MDS plot in between Hui population from China are in consistent with (yipping hao study). Same pattern of variation was also observed in heatmap plot between these 10 groups (Fig. 3), where Yunnan Hui showed most variations (green colour) followed by Sichuan, Shandong (brown colour) and Xinjiang (off brown colour) Hui. In PCA analysis (Fig. 4), we have observed 78.9% of variation on first two components (PC1 = 50.3% and PC2 = 28.6%). Interestingly in PCA, we have found five clusters, three clusters on upper half of plot while two clusters in lower half of cluster. Yunnan Hui clustered on extreme upper lift side of plot while Xinjiang Hui in the middle lift side, and five Hui groups from Qinghai, Gansu, Ningxia, Henan and Shaanxi formed big cluster on the upper right side of plot. Hui population from Liaoning formed its own cluster on the lower right side of plot while Sichuan and Shandong Hui formed a cluster in the lower middle lift side of the plot. Results of pairwise Fst, MDS plot, heatmap, interactivity test (Supplementary Fig. 2) and PCA showed that there are significant paternal genetic differences among 10 Hui ethnic groups across China and these results are supplement to [12].

To determine the precise structure of the Hui population, a median-joining network from Y-SNPs and Y-STRs was constructed. Most haplogroup substructures were generally extended, especially in O1 and O2. O2 and R1a's short-distance and star-like network structures showed a recent population expansion in Hui populations [34, 35]. Several studies have reported that the majority of Hui individuals carry Y-chromosomal haplogroup O-M175, with sub-haplogroups O1a-M119 and O2a1b1-M7 being the most common. These haplogroups are also common in other East Asian populations, particularly among the Han Chinese, and suggest a predominantly East Asian paternal origin for the Hui population [36–38]. However, the presence of other haplogroups such as J-M267, which is common in Central and West Asian populations, and E-M78, which is common in North African and West Asian populations, suggest that the Hui population also has a significant paternal genetic contribution from these regions [38, 39]. Historical records suggest that the Hui population has a complex history of migration and assimilation, with ancestral roots in Central Asia, the Middle East, and Southeast Asia [40, 41]. This may have contributed to the diverse genetic background of the Hui population, including their Y-chromosomal haplogroup distribution.

### Comparison of Hui populations with other Chinese populations

**Fig. 5:**
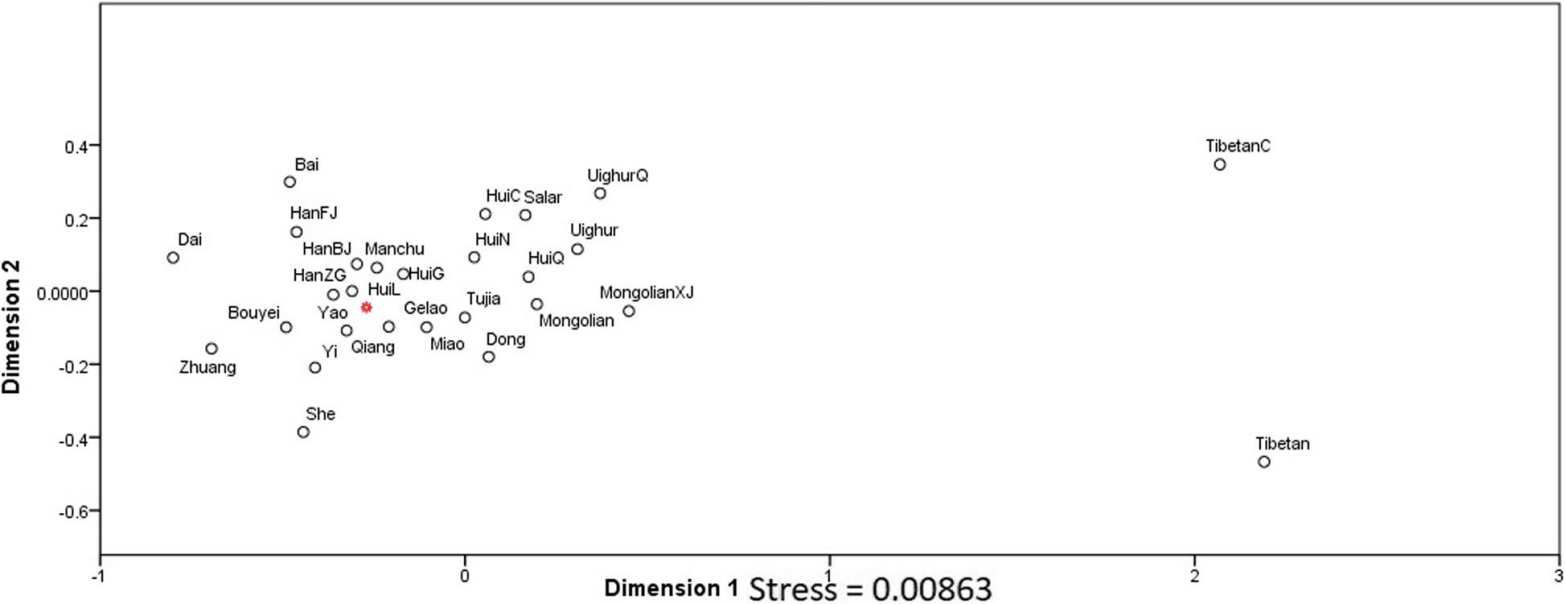
Two-dimensional plot from multi-dimensional scaling analysis of Rst-values based on PPY-23 haplotypes for Hui and other 25 Chinese populations

To investigate the relationship between Hui populations and other Chinese populations, pairwise Rst values were calculated (Supplementary table 6). The results showed that the Han population from Beijing exhibited the closest affinity to the Hui population, with a Rst value of 0.0005. The Manchu population from Liaoning followed closely behind with a Rst value of 0.0011. In contrast, the Tibetan population from Aba had the most distant affinity with the Hui population, with a Rst value of 0.3849. Another Tibetan population from Chamdo, China also had a distant affinity, with a Rst value of 0.3629. To further analyze the relationship between these populations, an MDS plot (Fig. 5) was generated using the Rst values. The plot showed that the Hui population was located on the lower right side of the plot, along with Yao, Qiang, and Gelao populations. The Han, Manchu, Dai, and Bai populations were situated on the upper left side, while the Uyghur, Salar, and Tibetan populations were on the upper right side. The Dong population was located on the lower right side of the plot. A heatmap analysis (Supplementary Fig. 3) was also conducted to compare the Hui population with other Chinese populations. The analysis revealed two main clusters, with cluster one further divided into four subclusters. The Hui populations were placed in the third subcluster of the first cluster. The Hui population from Liaoning was located in the second main cluster and the fifth subcluster along with the Han and Gelao populations. Results of PCA (Supplementary Fig. 4) showed 68.2% of variations among Chinese populations and 37.7% variations were at first component while rest were at second component. These results are also in accordance with MDS and heatmap. Overall, these results suggest that the Hui population is closely related to the Han and Manchu populations, but is distantly related to the Tibetan population. The MDS plot and heatmap analysis provide a visual representation of the relationships between the Hui population and other Chinese populations.

## Conclusion

In conclusion, our study has provided important insights into the genetic diversity and differences among Hui populations in China. Our results have shown that the Liaoning Hui population has a high level of genetic diversity and is distinct from other Hui populations in China. We have also observed significant paternal genetic differences among Hui populations across different regions of China. Additionally, our findings have revealed the relationship between Hui populations and other Chinese populations, with the closest affinity observed between the Hui and Han populations. The median-joining network analysis has highlighted the subhaplogroups that are characteristic of different geographical regions within the Hui population. Our study has limitations that need to be considered. We focused only on the genetic information passed down from fathers, so our understanding of the overall genetic diversity of the Hui population is partial. Future studies should include other genetic markers and consider both parents. Looking at haplogroups alone doesn't provide detailed information about the Hui population's history, migrations, or interactions with other groups. Additional analysis methods could shed light on these aspects. Moreover, our study only considered genetics, while culture and social factors are also important for understanding the Hui population. Future research should adopt a multidisciplinary approach. Despite these limitations, our study offers valuable insights into the genetic diversity and relationships among Hui populations in China, laying the foundation for further research to address these limitations and gain a more comprehensive understanding of the Hui people's genetic history and cultural identity.

### Supplementary Information

**Additional file 1:** **Supplementary Table S1.** The primer sequence of the Basic SNaPshot assay and 5 higher resolution SNaPshot assays for Y Haplogroups.

**Additional file 2:** **Supplementary Table S2.** Y-SNPs and Y-STR haplotypes and haplogroup information of 282 Hui population from Liaoning PR China.

**Additional file 3:** **Supplementary Table S3.** Allele range and frequencies of 26 Y STRs in 282 Hui population from Liaoning PR China along with forensic parameters.

**Additional file 4:** **Supplementary Table S4.** Pairwise Fst values between Hui population from Liaoning PR China and nine other Hui populations across China.

**Additional file 5:** **Supplementary Table S5.** Analysis of Molecular Variance (AMOVA) using Y SNPs and Y STRs between Hui population from Liaoning PR China and nine other Hui populations across China.

**Additional file 6: Supplementary Table S6.** Pairwise Rst values (below diagonal) and P values (above diagonal) for Hui population from Liaoning and other relevant population across China.

**Additional file 7: Supplementary Figure 1. **Phylogenetic tree showing Y-haplogroups found in the ten Hui populations.

**Additional file 8: Supplementary Figure 2. **Interactivity test between Hui population from Liaoning PR China and nine other Hui populations across China.

**Additional file 9: Supplementary Figure 3. **Heatmap analysis between Hui population from Liaoning and other relevant population across China.

**Additional file 10: Supplementary Figure 4. **Principal component analysis between Hui population from Liaoning and other relevant population across China.

## Data Availability

All data generated or analyzed during this study are included in this published article and its supplementary information files.
