# Supplementary figures and images for: Unraveling the paternal genetic structure and forensic traits of the Hui population in Liaoning Province, China using Y-chromosome analysis
Source: BMC Genomics. 2023 Nov 17;24:691. doi: 10.1186/s12864-023-09774-8 (PMC10655310; doi:10.1186/s12864-023-09774-8)

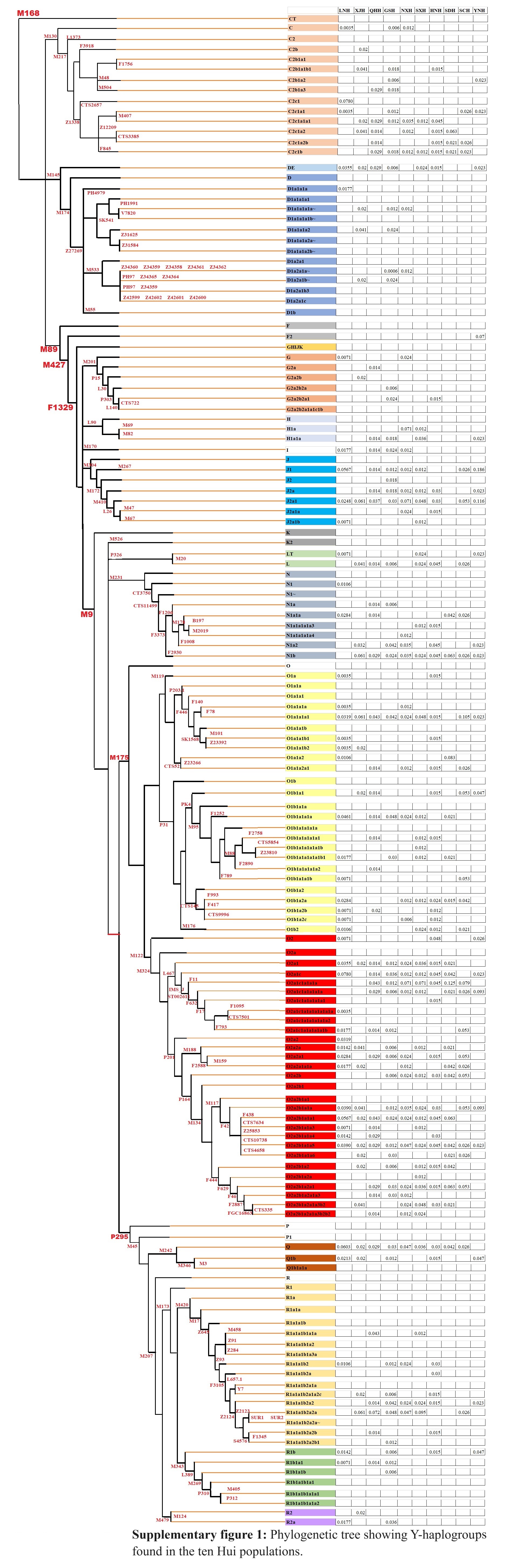

Supplement: Supplementary file 7 — Additional file 7: Supplementary Figure 1. Phylogenetic tree showing Y-haplogroups found in the ten Hui populations. [file 12864_2023_9774_MOESM7_ESM.jpg]

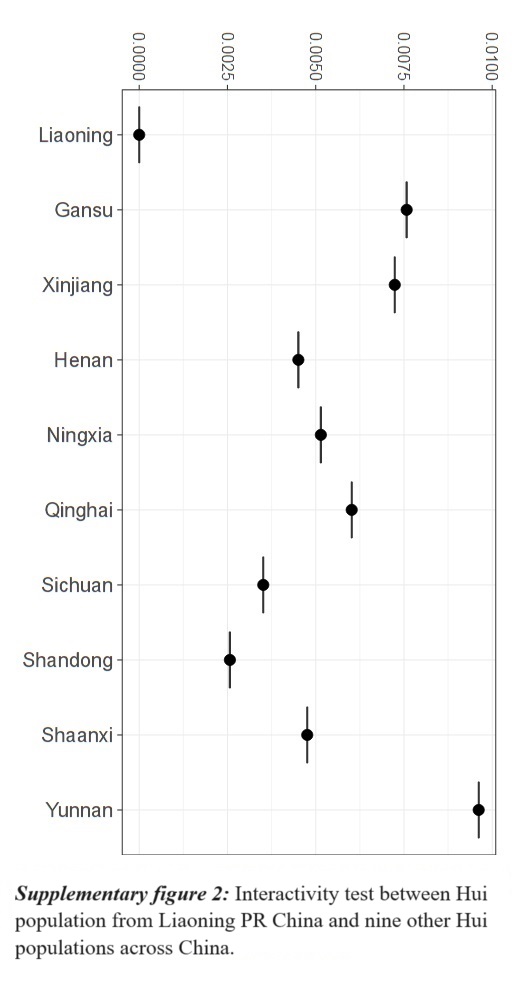

Supplement: Supplementary file 8 — Additional file 8: Supplementary Figure 2. Interactivity test between Hui population from Liaoning PR China and nine other Hui populations across China. [file 12864_2023_9774_MOESM8_ESM.jpg]

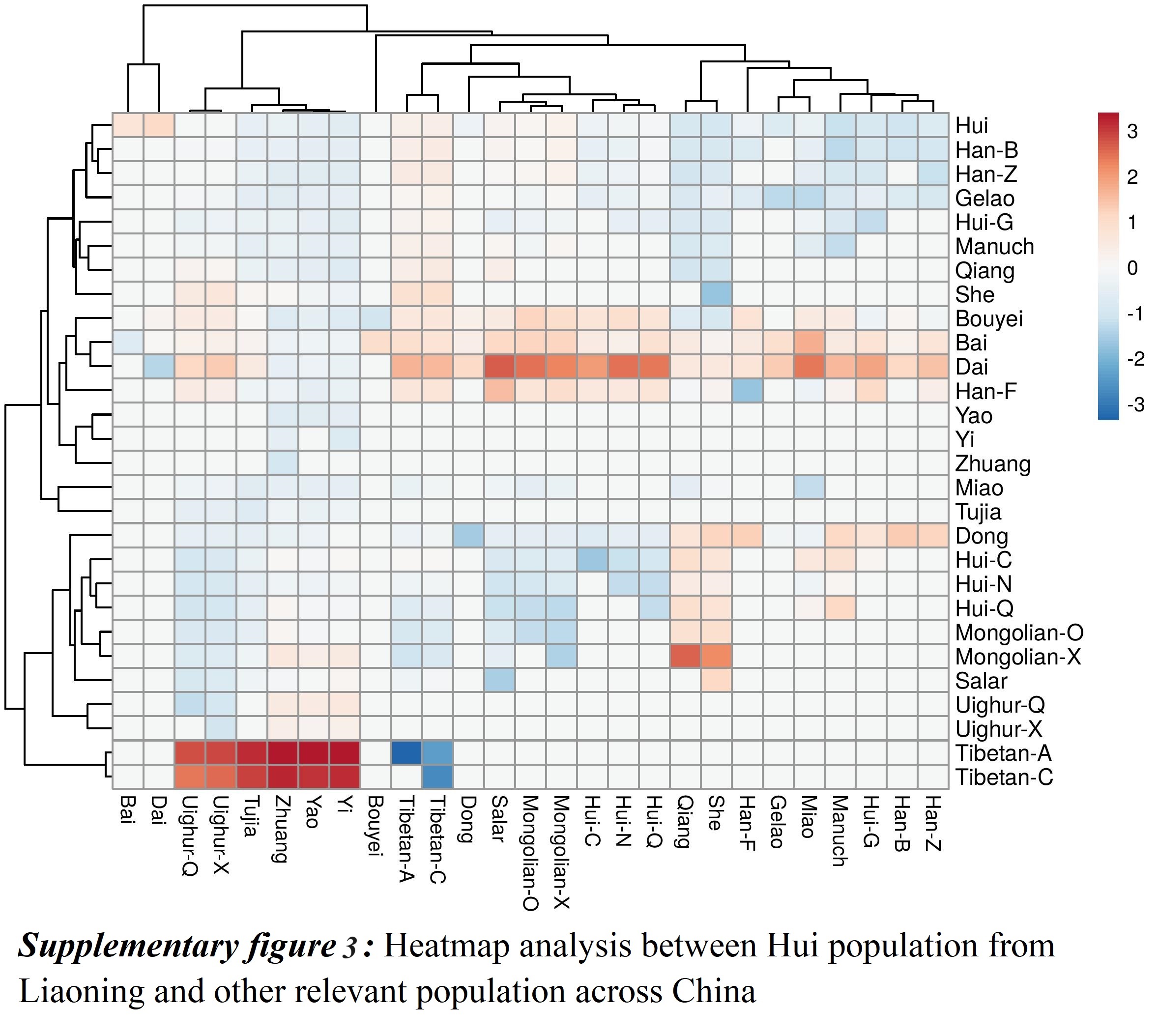

Supplement: Supplementary file 9 — Additional file 9: Supplementary Figure 3. Heatmap analysis between Hui population from Liaoning and other relevant population across China. [file 12864_2023_9774_MOESM9_ESM.jpg]

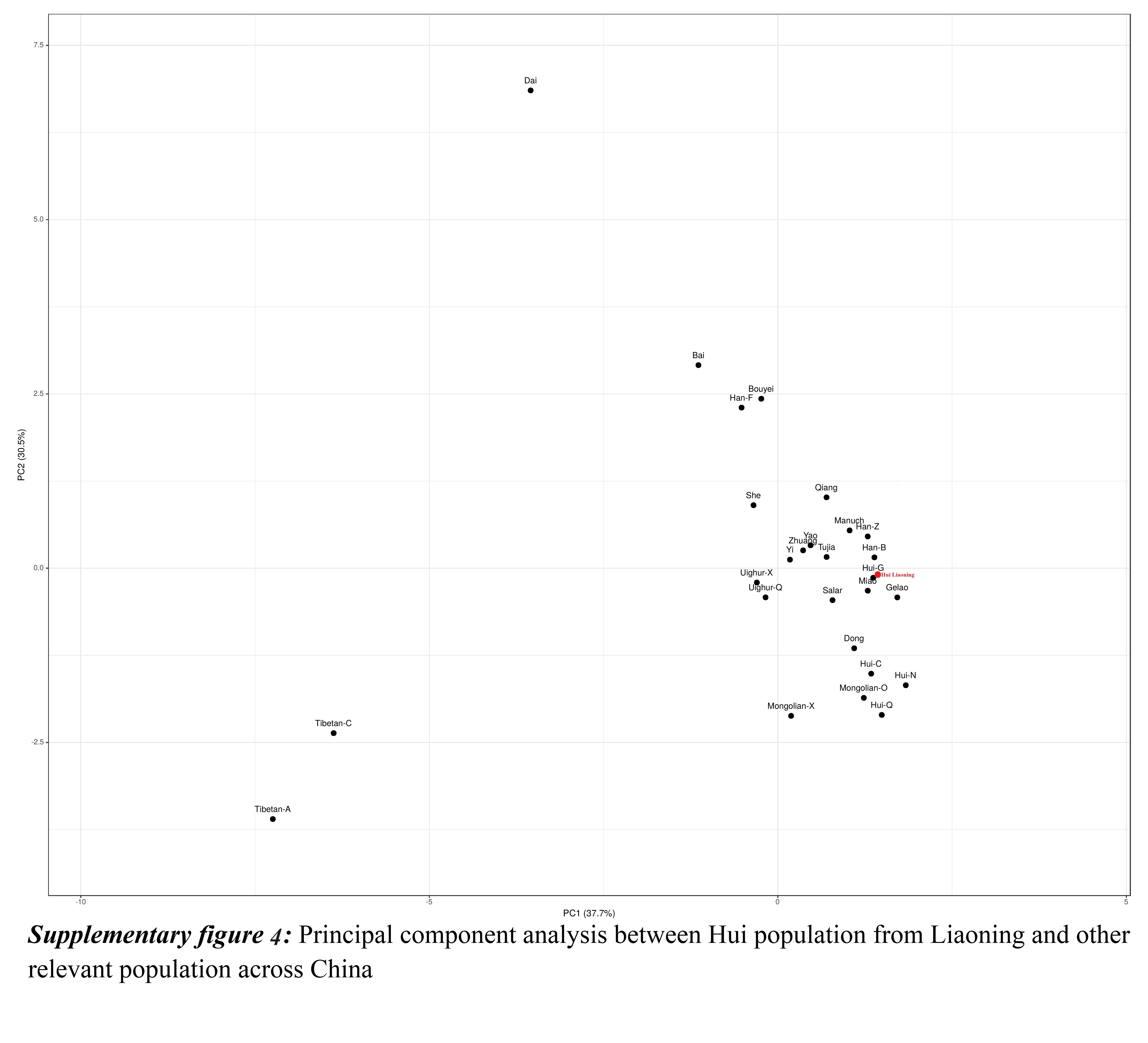

Supplement: Supplementary file 10 — Additional file 10: Supplementary Figure 4. Principal component analysis between Hui population from Liaoning and other relevant population across China. [file 12864_2023_9774_MOESM10_ESM.jpg]
